# Pedicled vascularized fibular graft with Ilizarov external fixator for reconstructing a large bone defect of the tibia after tumor resection

**DOI:** 10.1007/s10195-013-0225-3

**Published:** 2013-02-16

**Authors:** Yousuf M. Khira, Hamed A. Badawy

**Affiliations:** Orthopedic Department, Faculty of Medicine, Zagazig University, Zagazig, Egypt

**Keywords:** Pedicled, Vascularized, Fibula, Bone defect, Tumor

## Abstract

**Background:**

Large bony defects in the middle or distal third of the tibia resulting from surgical resection of malignant bone tumors present a difficult reconstructive challenge. Various methods of reconstruction are available, such as allografts, vascularized fibular graft (either free or pedicled), or endoprothesis replacement for distal defects.

**Materials and methods:**

Twelve patients—eight males and four females with mean age of 18 years at operation (range 14–25 years)—with malignant bone tumors of the tibial shaft were selected as candidates for wide resection of the tumor and reconstruction of the bony defect by ipsilateral vascularized fibular graft based on the peroneal vessels. Preoperative staging studies, including plain radiography, local MRI, isotopic bone scan, and chest CT, were done for every patient before biopsy. Ilizarov external fixation was then applied in all cases. The average length of the bony gap bridged was 14.5 cm (13–16.5 cm) and the mean length of the harvested graft was 16.3 cm (15–18 cm). The average operation time was 7.5 h (5.5–9.5 h).

**Results:**

The mean follow-up period was 38 months (range 32–52 months). Bony union at the proximal and distal ends of the fibula occurred in nine patients (75 %) and at a mean time of 5.5 months (range 4.5–8 months). Graft hypertrophy occurred in all patients. The mean percentage of hypertrophy was 95 % (range 80–160 %). The mean MSTS functional score was 84 % (range 80–92 %). A leg length discrepancy of 2 cm was reported in two patients and was managed using a shoe lift.

**Conclusion:**

Reconstruction of bony defects of the middle or distal tibia after bone tumor resection using pedicled vascularized fibula is a useful limb salvage procedure. The procedure can be performed relatively quickly and inexpensively and has a low rate of late complications. It leads to a good outcome regarding the union, hypertrophy, and function.

## Introduction

Bone sarcomas in children and young adults are rare but highly malignant. The most frequent histological types are osteosarcoma and Ewing’s sarcoma. The treatment method for these tumors in the extremities consists of chemotherapy and surgical tumor resection [1, 2]. This combined treatment has led to a significantly increased survival rate since it was introduced in the 1970s [3]. The five-year survival rate in patients with non-metastatic disease is above 70 %. The introduction of chemotherapy combined with the development of imaging and surgical techniques has made it possible to perform bone tumor resections and limb-sparing surgery in more than 80 % of cases without incurring increased mortality [4, 5]. Large bony defects in the middle or distal third of the tibia resulting from surgical resection of malignant bone tumors present a difficult reconstructive challenge. Various methods are available to the reconstructive surgeon, such as allografts, vascularized fibular graft (either free or pedicled), or endoprothesis replacement for distal defects [6].

Depending on various parameters such as the anatomical location of the tumor and patient age, there are different reconstruction techniques that can be employed, each of which have their advantages and disadvantages. Allografting is a solution for bone loss after tumor excision. Drawbacks of allografting include: expense, irregular supply (especially in developing countries), and a high rate of complications, such as infection, fracture, delayed union, and the potential for late failure [3, 5].

Another method is endoprothetic reconstruction. When reconstructing the proximal tibia, the preferred method is modular prostheses; on the other hand, custom-made prostheses are suitable for diaphyseal and distal tibia reconstructions. Using this technique, limb salvage can be maintained in more than 80 % of patients 20 years after the primary reconstruction [7]. Complications of endoprosthetic reconstruction of the distal tibia include infection, inadequate soft tissue coverage, and talar collapse [4, 8].

Nonvascularized autogenous bone grafts may lead to nonunion and repeated fractures and they do not exhibit compensatory hypertrophy, so they are not suitable for large defects following malignant bone tumor resection [9].

Vascularized fibular graft based on the peroneal vessels has been recognized to be a useful technique for reconstructing large tibial bone defects following tumor resection, especially for the middle or distal part [10, 11]. Because the fibula is a straight long bone and consists of dense cortical bone along its length, it provides rapid union in a short time between the graft and the recipient bone. This, coupled with its triangular cross-section, allows it to resist angular and rotational stresses. After bone healing has been established, the vascularized fibular graft can be seen to hypertrophy to a varying degree in response to mechanical forces [12].

In the prospective study described in the present paper, we investigated the results of resecting an isolated diaphyseal bone tumor of a long bone and performing reconstruction using pedicled vascularized fibula grafts in twelve young patients.

## Materials and methods

All patients gave their informed consent prior to being included in the study.

The study was authorized by the local ethical committee and was performed in accordance with the ethical standards of the 1964 Declaration of Helsinki, as revised in 2000.

Twelve patients diagnosed with malignant bone tumors of the tibial shaft underwent wide resection of the tumor and reconstruction of the bone defect using ipsilateral vascularized fibular graft based on the peroneal vessels in the period from 2005 to 2010. There were eight males and four females ranging from 14 to 25 years old with a mean age of 18 years at operation who had malignant bone tumors of the shaft of tibia. Plain radiography, MRI of the affected limb, isotopic bone scan, and chest CT were done for every patient in the study, followed by biopsy from the lesion. Osteosarcoma was diagnosed in in eight patients and Ewing’s sarcoma in the other four. MR angiography or angiography was done routinely to exclude vascular invasion. According to the Enneking staging system [13], nine patients had stage IIB and three had stage IIA. Neoadjuvant chemotherapy was given to all patients with osteosarcoma and Ewing’s sarcoma (Table 1).

**Fig. 1:**
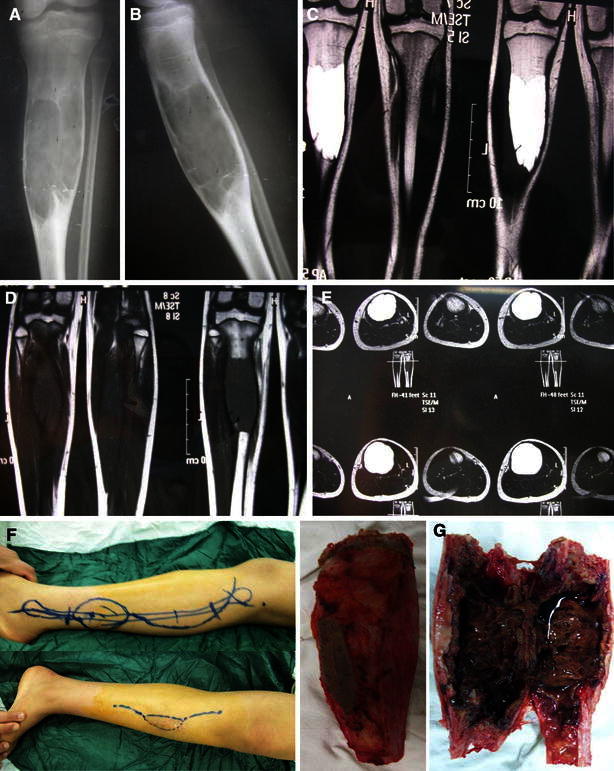
Operative technique for tumor resection and planning for fibula harvesting (18-year-old female patient with bone sarcoma of the tibia). **a**, **b** Preoperative plain X-ray; **c**–**e** preoperative MRI for diagnosis and staging; **f**, **g** tumor mass after resection

Patients were considered candidates for wide resection of their tumors and reconstruction of the bony defect if preoperative studies showed that satisfactory surgical margins could be achieved without resecting the ipsilateral fibula or compromising its pedicle. Local wide resection with free safety bony and soft tissue margins was achieved in all cases. Fibula was harvested as an osteoseptocutaneous graft through a posterolateral approach with a skin paddle attached to the fibula by the posterior intermuscular septum. This septum contained septal perforator vessels that originated from the peroneal vessels and supplied the skin paddle (Fig. 1). Tumor resection was done using a separate approach. The skin paddle had a dual function. First, it acted as an indicator of the viability of the fibular graft postoperatively. Second, it provided tension-free closure of the recipient site, especially when a large skin area was excised with the biopsy scar or there was partial skin necrosis resulting from the elevation of large flaps during tumor resection. The skin flap was designed with its center located at the junction of the middle and distal thirds of the leg and its longitudinal axis coinciding with the posterior border of the fibula.

The site of fibular osteotomy was determined, leaving at least 6 cm of the fibula distally to maintain ankle stability and 3–4 cm proximally to maintain lateral stability of the knee. Even when only a short segment of bone is needed, it is desirable to remove the whole length of the fibula to facilitate peroneal pedicle dissection.

**Fig. 2:**
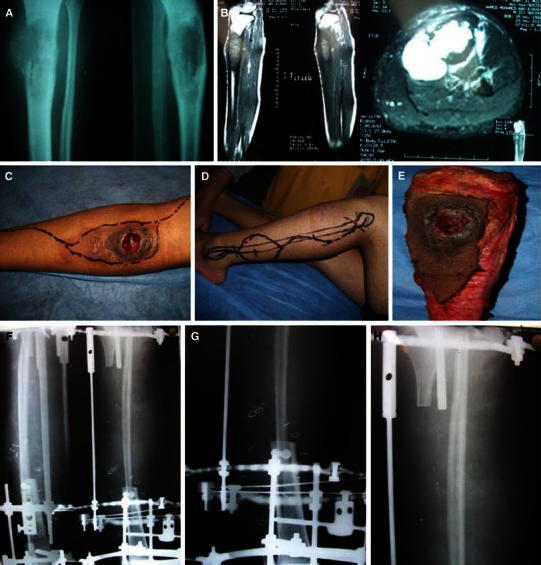
Male patient 22 years old with bone sarcoma of the tibia. **a** Preoperative plain X-ray; **b** preoperative MRI for diagnosis and staging; **c**, **d** intraoperative photos showing skin incision planning for tumor resection and fibula harvest; **e** intraoperative photo of tumor mass after resection; **f** plain postoperative X-ray with Ilizarov fixator applied; **g, h** plain X-ray taken three months postoperatively

The inset of the harvested fibular flap was trimmed to the desired length taking into consideration the amount needed for bone doweling, with attention paid to the axial and rotational alignment of the limb (done under an image intensifier). Both ends of the fibula were fixed to the recipient bones by intramedullary doweling with transfixing screws on each side. Skin incision for tumor resection was carefully designed to include the biopsy site and any previous operative scars, and to allow direct exposure of the major neurovascular bundles. This was mostly done through a separate posteromedial approach to facilitate exposure of the posterior tibial neurovascular bundle. On the other hand, isolation of the anterior tibial bundle was done through the approach to the fibula. After isolating the major neurovascular bundle, the tumor was excised with a wide margin at a distance of at least 3 cm from the intramedullary or cortical extent of the tumor as determined by preoperative MRI. Depending on the extent of the tumor, resection was intercalary (sparing the joints above and below the tumor). Ilizarov external fixation was then applied in all cases (Fig. 2). The average length of the bony gap bridged was 14.5 cm (13–16.5 cm) and the mean length of the harvested graft was 16.3 cm (15–18 cm). The average operation time was 7.5 h (5.5–9.5 h).

In the early postoperative phase, graft perfusion was evaluated by monitoring the skin flap paddle for color, temperature, turgor, capillary refill, and bleeding on pinprick. Healing and graft hypertrophy was evaluated by examining repeated plain X-rays. Isotope scanning was used to evaluate graft perfusion, local recurrence, and bone metastases in eight cases. Clinical outcome was assessed according to the Musculoskeletal Tumor Society (MSTS) score [14], which evaluates pain, functional activity, emotional acceptance, need for external support, walking ability, and gait.

**Fig. 3:**
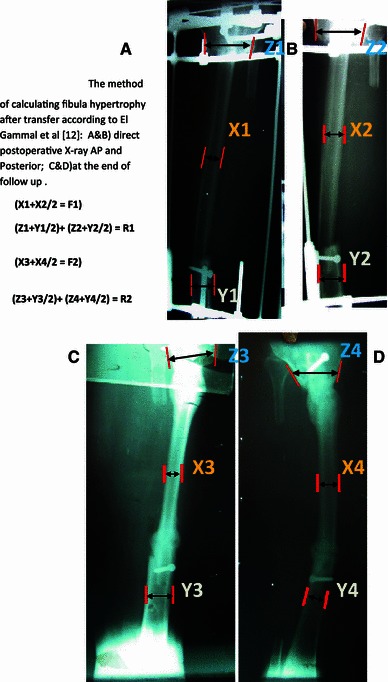
The method used to calculate the fibular hypertrophy in relation to host bone (labeled)

Bony union of the graft proximally and distally was assessed and defined as uninterrupted external bony borders between the fibular graft and recipient bone. The amount of hypertrophy that had developed in the fibula at the time of final follow-up was calculated according to the following formula used by El-Gammal et al. [12] (see also Fig. 3):where *F*_1_ = mean fibular graft anteroposterior and lateral width at the midpoint postoperatively, *R*_1_ = mean recipient bone anteroposterior and lateral width at a fixed point away from the graft–host junction postoperatively, *F*_2_ = mean fibular graft anteroposterior and lateral width at the midpoint at follow-up, and *R*_2_ = mean recipient bone anteroposterior and lateral width at a fixed point away from the graft–host junction at follow-up.

**Fig. 4:**
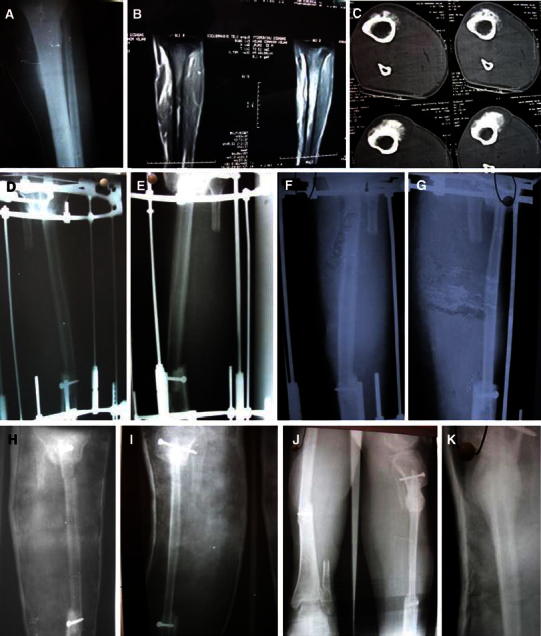
Male 25-year-old patient with bone sarcoma of the tibia: **a** preoperative plain X-ray; **b**, **c** preoperative MRI for diagnosis and staging; **d**, **e** postoperative X-ray with Ilizarov external fixator after fixation of the fibula with two interfragmentary screws; **f**, **g** plain X-ray taken six months postoperatively; **h**, **i** stress fracture after the Ilizarov fixator and above-knee cast was applied; **j** X-ray showing union of stress fracture; **k** plain X-ray taken 18 months postoperatively, showing fibula hypertrophy

When radiographs showed signs of proximal and distal union, the external fixator was removed and an above-knee cast was then applied (Fig. 4). Gradual weight bearing was allowed with radiological evidence of union and excluding stress fractures. Full weight bearing without a cast was allowed when graft hypertrophy matched or approximated the size of the shaft of the tibia. Postoperative chemotherapy was given to seven cases. One of these seven cases did not respond to the chemotherapy, so radiotherapy was given, but unfortunately this patient had local recurrence and above-knee amputation was performed. All of the results provided below are given as the mean and the total range.

**Table 1:** Data for all of the patients included in this study

Pt. no.	Age	Sex	Histology	Local recurrence	Metastasis	Bone defect (cm)	Fibula length (cm)	Operative time (h)
1	14	Male	Ewing’s sarcoma	No	No	13	14.5	6
2	18	Female	Ewing’s sarcoma	No	No	15	16.5	7
3	15	Male	Osteosarcoma	No	No	15	17	5.5
4	25	Male	Osteosarcoma	No	No	14	15.5	8
5	22	Male	Osteosarcoma	No	No	15	16.5	9.5
6	16	Female	Ewing’s sarcoma	No	No	16	18	8
7	14	Female	Osteosarcoma	No	No	13	15	7.5
8	16	Male	Ewing’s sarcoma	No	No	14.5	16	7
9	18	Male	Osteosarcoma	No	No	14	15.5	7.5
10	20	Male	Osteosarcoma	Yes	No	16	17.5	8
11	19	Male	Osteosarcoma	No	Yes	15	16	9.5
12	19	Female	Osteosarcoma	No	Yes	16.5	18	7

**Table 2:** Results

Pt. no.	Follow-up period (months)	Bone healing (months)	MSTS score (%)	Stress fracture and treatment	Other complications
1	48	8	84	No	No
2	42	4.5	85	No	No
3	45	4.5	80	No	No
4	52	5	92	Yes (above-knee cast)	Ankle valgus Supramalleolar osteotomy
5	40	5	90	No	No
6	38	5.5	82	Yes (in cast)	Sciatic nerve palsy
7	36	4.5	84	No	No
8	50	5	85	No	No
9	36	5	80	No	No
10	34	6	82	No	No
11	35	8	80	Yes (plate fixation)	No
12	32	5	84	No	Sciatic nerve palsy

**Fig. 5:**
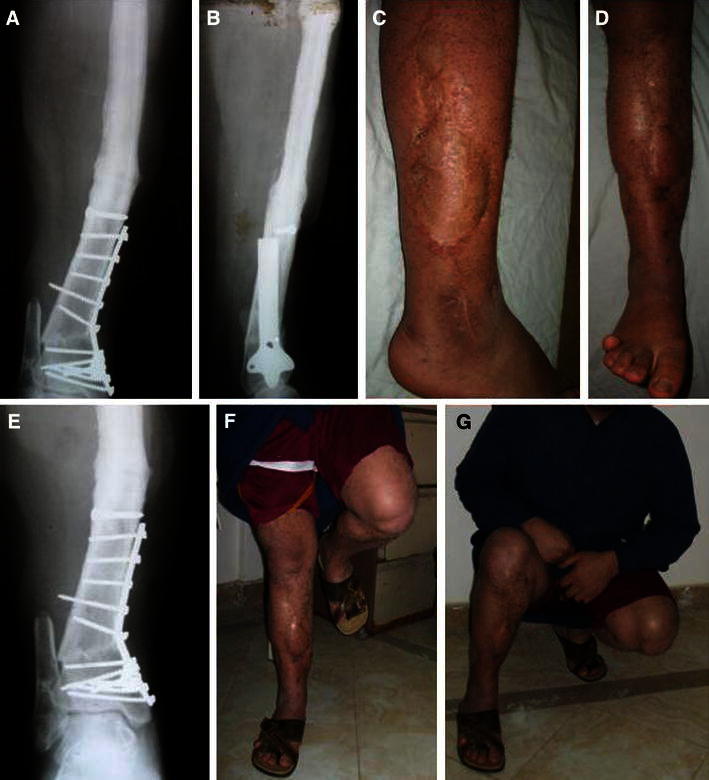
Clinical and radiological outcome of 25-year-old male patient three years after the operation. **a**, **b** Plain X-ray taken six months after supramalleolar osteotomy for valgus angulation (note also the hypertrophy of the fibula); **c**, **d** photo of the leg after skin healing of incisions and flaps; **e** X-ray taken one year after the osteotomy, showing good union; **f**, **g** photos showing weight bearing and movement of the patient at the end of the follow-up period

## Results

The mean follow-up period was 38 months (range 32–52 months). Bony union at the proximal and distal ends of the fibula without complications occurred in nine patients (75 %) at a mean time of 5.5 months (range 4.5–8 months). Graft hypertrophy occurred in all patients. The mean percentage of hypertrophy was 95 % (range 80–160 %). The mean time taken for the fibula to double in size (based on ten cases) was 24 months (range 18–36 months). Failure of hypertrophy did not occur in any of the patients; however, it was relatively delayed in two cases. One of these two cases had nonunion at the distal junction with ankle deformity, and required grafting of the ununited end and internal fixation with plate and screws. The other case needed only a bone graft and cast. The two cases finally achieved union at a mean time of five months after revision (Table 2).

The mean MSTS functional score was 84 % (range 80–92 %). Leg length discrepancy was reported in two cases. The shortening was about 2 cm and the patients were managed using a shoe lift.

Complications reported in the study were as follows. Stress fracture of the transferred fibula was seen in three cases (25 %). One case had asymptomatic stress fracture early after union and before the development of hypertrophy. It was detected on follow-up radiographic examination and had healed within three months upon using an above-knee cast for one and half months and a walking cast for another 45 days. The other two patients (neither of whom had a history of trauma) had stress fractures in hypertrophied fibulae after cast removal. One of them needed a bone graft and was splinted in an above-knee cast for two months, followed by orthosis to protect the bone for six months; the other was internally fixed by plating and bone graft.

A slight angulation at the junction of the proximal or distal end of the transferred fibula was seen in five cases. The degrees of angulation were minor and did not require surgical intervention in four of these cases, but ankle valgus was severe in one case and needed supramalleolar wedge osteotomy and plate fixation (Fig. 5).

Infection occurred only as a pin-tract infection, and was controlled with oral antibiotics and repeated dressing, but there was no deep infection in any of these patients. Oral cephalosporin antibiotics were used routinely in seven cases that required postoperative chemotherapy.

Common peroneal nerve palsy was detected in two patients with proximal tibial tumors in the immediate postoperative period. Both of them completely recovered within 6 months.

One case developed local recurrence 30 months after primary resection, and pulmonary metastasis was discovered in two patients after 25 and 28 months, respectively. The patient with the local recurrence had above-knee amputation. The patients who developed pulmonary metastasis were referred to chemotherapy and died of disease progression.

## Discussion

Segmental bone defects continue to be one of the most challenging and difficult problems for any orthopedic surgeon. Different reconstructive techniques have been described for such defects, including nonvascularized cancellous bone grafts, bone transport using the Ilizarov external fixator, and vascularized bone grafts. However, the ideal bone graft for bone defects larger than 6 cm is the vascularized bone graft with or without autograft and allograft [16–18].

The vascularized fibula flap has been widely used to reconstruct bone defects, providing up to 25 cm long high-density straight cortical bone with a good vascular pedicle [19]. However, there are disadvantages to free vascularized fibula transfer: it requires another team skilled in this microvascular technique, involves a long operative time of 6–10 h, utilizes substantial hospital resources, produces moderate to severe pain at the donor site, and leads to delayed wound healing [20]. It also sacrifices a major vessel of the lower extremity, requires immediate postoperative monitoring of circulation to the graft, and its success is questionable in areas that have undergone previous irradiation or severe trauma [21]. In contrast, ipsilateral vascularized fibula transposition is a technique that also has some limitations, as it is not suitable for very proximal or distal tibia defects because the main blood supply of the fibula comes from a branch of the peroneal artery which enters the posterior middle third 6–7 cm below its origin [22]. Second, the length of the fibular pedicle should be sufficient for transfer. When compared to free vascularized fibula transfer, pedicled fibula transfer has the advantages of a shorter operative time as well as preservation of the vessel pedicle and some of the soft tissues around the bone. In addition, microsurgical experience is not necessary.

Few studies have evaluated the functional results after reconstructing bone defects with vascularized fibula grafts following tumor resection. An average MSTS score of 24 (80 %) was obtained in the study conducted by Michael et al. [6], whereas the MSTS score was on average between 26.5 and 30 in another study performed by Germain et al. [21], which very briefly presented the results for 78 children. However, the MSTS scores in this study ranged from 24 to 27.5 points and were comparable with those published by El-Gammal et al. [12] and Rose et al. [23]. Vascularized fibular graft has the ability to develop hypertrophy when subjected to mechanical loading. Although hypertrophy has been noted by many authors, it has only rarely been quantified [24].

De Boer et al. [25] introduced a graft hypertrophy index: (G2/R2 − G1/R1)/G1R1, where G1 is the graft diameter at the proximal junction during the operation, R1 is the host bone diameter at the proximal junction, G2 is the graft diameter at the proximal junction at the most recent follow-up, and R2 is the host bone diameter at the proximal junction at the most recent follow-up. A positive index value confirms hypertrophy. However, Amr et al. [26] stated that this hypertrophy index is unreliable because it is a measure of fibular hypertrophy in relation to recipient bone. False results are obtained when callus formation at the recipient bone is more abundant at the proximal end of the fibula. In addition, hypertrophy of the fibula is not homogeneous at both the upper and the lower ends of the graft.

Falder et al. [15] measured hypertrophy and expressed it as a percentage of the difference between the original fibula width and the final fibula width. In this study, we used the calculation method of Falder et al., and we found that the mean percentage of hypertrophy was 95 % (range 80–160 %) and the mean time taken for the fibula to double in size (based on seven cases) was 24 months (range 18–36 months). The amount of hypertrophy has been found to increase with time since surgery, and it exceeded the diameter of the recipient bone for the whole length of the fibula bone in two cases. This may be related to the large amounts of walking performed by young patients, which exposes the fibula to continuous mechanical stresses (the main factor promoting the development of hypertrophy).

Stress fracture of a vascularized fibular graft is a complication that has been reported by many authors, even in early hypertrophied bone [11, 16]. Repetitive mechanical loading which exceeds the bone strength is the cause of this complication. The incidence of stress fracture in this study was 25 %. One case had a stress fracture in early hypertrophied bone, which demonstrates why care should be taken during walking and adequate support of the fibula is required until sufficient hypertrophy has occurred to allow safe full weight bearing.

Achieving stability after fibular transposition is the key to facilitating union. Rapid union between the graft and the recipient bone and the development of hypertrophy contributes to limb stability and the achievement of good functional results. Ozaki et al. [27] state that fixation with a long plate and several screws is better than minimal osteosynthesis, whereas Ebied et al. [10] recommended fixation with screws, K-wires, or cerclage wires and external fixation of the limb in a plaster cast, based on the results obtained for their series. The method of fixation used in this study was external fixation in all cases, with added proximal and distal screws in graft junctions. In this study, common peroneal nerve palsy was detected in two patients with proximal tibial tumors during the immediate postoperative period. Both of them completely recovered within six months. This was probably due to overtraction of the nerve.

Reconstruction of bony defects of the middle or distal tibia after bone tumor resection using pedicled vascularized fibula is a useful limb salvage procedure. The procedure can be performed relatively quickly and inexpensively, and has a low rate of complications. It leads to a good outcome regarding the union, hypertrophy, and function.
